# Handling the phosphorus paradox in agriculture and natural ecosystems: Scarcity, necessity, and burden of P

**DOI:** 10.1007/s13280-017-0968-9

**Published:** 2017-11-20

**Authors:** Peter Leinweber, Ulrich Bathmann, Uwe Buczko, Caroline Douhaire, Bettina Eichler-Löbermann, Emmanuel Frossard, Felix Ekardt, Helen Jarvie, Inga Krämer, Christian Kabbe, Bernd Lennartz, Per-Erik Mellander, Günther Nausch, Hisao Ohtake, Jens Tränckner

**Affiliations:** 1 Department of Soil Science, Faculty for Agricultural and Environmental Sciences, University of Rostock, Justus-von-Liebig Weg 6, 18059 Rostock, Germany; 2 Leibniz-Institut für Ostseeforschung Warnemünde, Seestraße 15, 18119 Rostock, Germany; 3 Landscape Ecology and Site Evaluation, University of Rostock, 18059 Rostock, Germany; 4 Forschungsstelle Nachhaltigkeit und Klimapolitik, Könneritzstraße 41, 04229 Leipzig, Germany; 5 Department of Crop Production, Faculty of Agricultural and Environmental Sciences, Justus-von-Liebig Weg 6, 18059 Rostock, Germany; 6 ETH Zurich, Research Station in Plant Sciences, Eschikon, 8315 Lindau, Switzerland; 7 Centre for Ecology & Hydrology, Wallingford, Oxfordshire OX10 8BB UK; 8 Leibniz Science Campus Phosphorus Research Rostock c/o, Leibniz Institute for Baltic Sea Research Warnemünde, Seestr. 15, 18119 Rostock, Germany; 9 P-REX Environment, Am Goldmannpark 43, 12587 Berlin, Germany; 10 Department of Soil Physics, Faculty of Agricultural and Environmental Sciences, University of Rostock, Justusvon-Liebig Weg 6, 18059 Rostock, Germany; 11 Department of Environment, Soils and Landuse, Teagasc, Johnstown Castle Environmental Research Centre, Johnstown Castle, Co. Wexford Ireland; 12 Baltic Sea Institute for Baltic Sea Research Warnemünde (IOW), Seestrasse 15, 18109 Rostock, Germany; 13 Phosphorus Atlas Research Institute, Waseda University, Wakamatsu-cho 2-2, Shinjuku-ku, Tokyo, 162-0056 Japan; 14 Water Management, Faculty of Agricultural and Environmental Sciences, Satower Strasse 48, 18059 Rostock, Germany

**Keywords:** Cropping system, Eutrophication, Fertilizer, Governance, P efficiency, Plant nutrition

## Abstract

This special issue of *Ambio* compiles a series of contributions made at the 8th International Phosphorus Workshop (IPW8), held in September 2016 in Rostock, Germany. The introducing overview article summarizes major published scientific findings in the time period from IPW7 (2015) until recently, including presentations from IPW8. The P issue was subdivided into four themes along the logical sequence of P utilization in production, environmental, and societal systems: (1) Sufficiency and efficiency of P utilization, especially in animal husbandry and crop production; (2) P recycling: technologies and product applications; (3) P fluxes and cycling in the environment; and (4) P governance. The latter two themes had separate sessions for the first time in the International Phosphorus Workshops series; thus, this overview presents a scene-setting rather than an overview of the latest research for these themes. In summary, this paper details new findings in agricultural and environmental P research, which indicate reduced P inputs, improved management options, and provide translations into governance options for a more sustainable P use.

## Introduction

Phosphorus (P) has been identified as a critical resource for the bioeconomy and for food security by the European Union (EU) and at the global scale (Cordell and White 2014). The biogeochemical P flow has been described as a “planetary boundary” which, in parts of the world, has already been exceeded (Carpenter and Bennett 2011; Steffen et al. 2015). Over the past few years, concern about the growing P ‘paradox‘(the simultaneous over-abundance of P impairing water quality, and the prospect of global scarcity of P for future agricultural production) has stimulated new convergence between P-security and water-quality research agendas (Cordell and White 2015; Jarvie et al. 2015; Nesme and Withers 2016). This convergence also reflects a growing recognition that improving societal efficiency in P use will be fundamental in addressing both sides of the P paradox (Jarvie et al. 2015; Withers et al. 2015). Increasing availability of datasets at the national, regional, and global scales, has stimulated new evaluation of the patterns in P stocks, flows, and stores in agricultural and urban systems; the fragmentation of the P cycle; and implications for water-quality impairment (Cordell and White 2015; Metson et al. 2015, 2016; Rowe et al. 2016; Sharpley et al. 2016; Worrall et al. 2016); and wider ecosystem services (MacDonald et al. 2016). These assessments are providing new insights into the disconnects and imbalances in P cycles, and the societal inefficiencies in P use, across broad spatial scales (Scholz et al. 2015; Scholz and Wellmer 2015). However, there are large spatial disconnects between the macroscale needs for more efficient P-resource management, and the local realities of P management at the farm-scale where decisions are made (Osmond et al. 2015; Sharpley 2016; Sharpley et al. 2016). Moreover, there is a growing recognition of the limits to which the results from macroscale assessments can be extrapolated to make recommendations for changes in agricultural policy and practice for improved P stewardship, and that it is vital that local, environmental, and socioeconomic realities and nuances are taken into account (Sharpley et al. 2016).

Research activities in response to these challenges have resulted in a number of international workshop and conferences series, such as the Sustainable Phosphorus Summits or International Phosphorus Workshops. The 8th International Phosphorus Workshop (IPW8) held in Rostock, Germany in September, 2016 launches this special issue of the journal *Ambio* in which selected contributions to the workshop are published. In this introductory overview article, we aim to summarize the recent scientific progress between IPW7 (Sharpley et al. 2015) and IPW8. The paper is concentrating on issues related to P-rich environments and countries, while the serious P-related issues globally—especially in low-income countries, with many farmers who can hardly afford P fertilizers and recovering technologies—have not been broadly discussed at the IPW8, and therefore are not adequately reflected in this *Ambio* special issue.

**Fig. 1:**
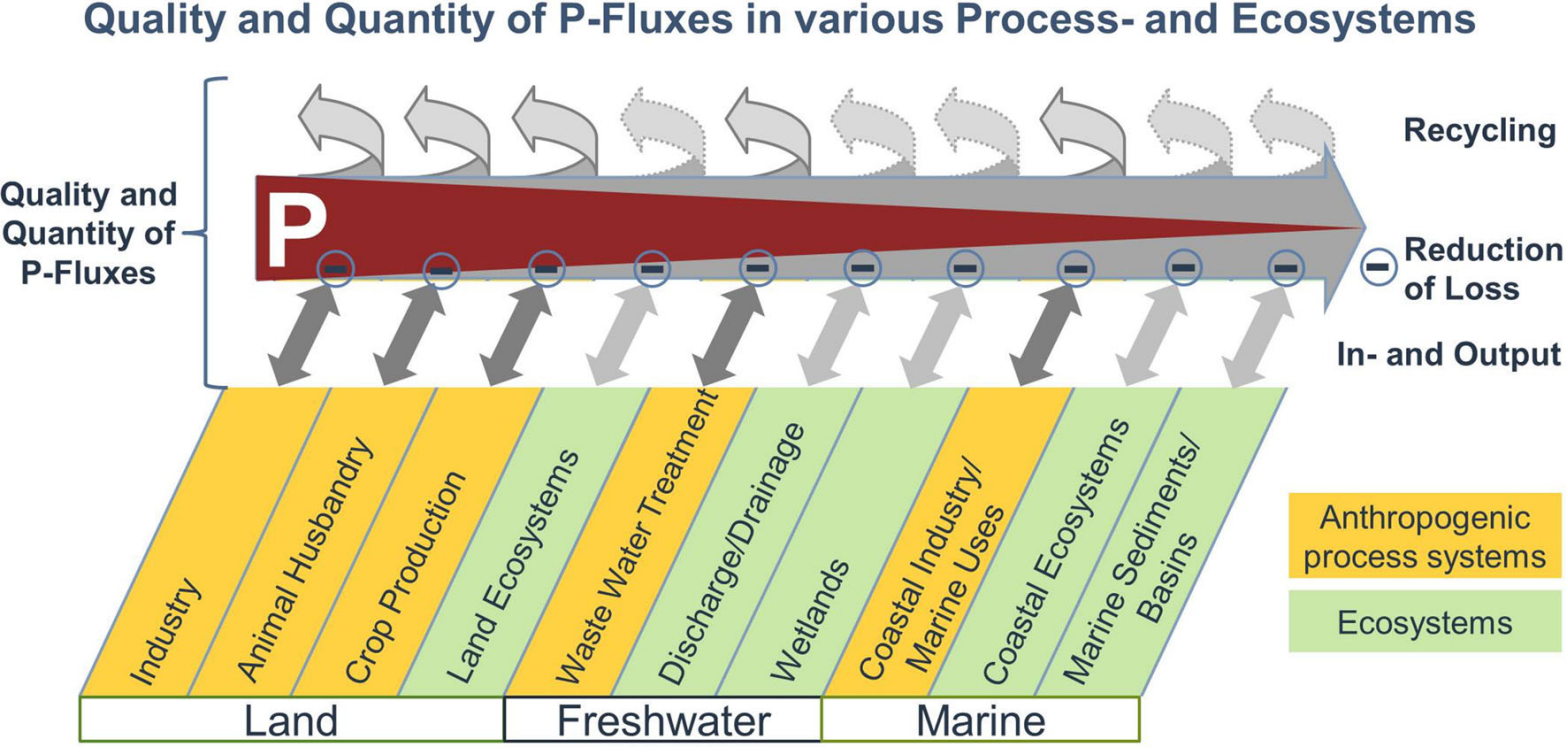
Schematic presentation of P fluxes through various process and ecosystems along which originally mined and processed P is diluted and distributed over increasingly large parts of the terrestrial and aquatic environments

In accordance with the structure of the IPW8-meeting, and the flow of P through various processes and ecosystems as visualized in Fig. 1, this overview article is subdivided into four themes: (1) sufficiency and efficiency of P utilization, especially in animal husbandry and crop production; (2) P recycling: technologies and product applications, (3) P fluxes and cycling in the environment; and (4) P governance. For each of these themes, we evaluated the workshop contributions and the recent literature for (i) the most significant results of the last three years, (ii) areas in which most progress has been made and new research trends, and (iii) areas in which more research and funding programs and/or in which political/legislative actions are required. This paper summarizes the outcome of session reports and expert discussions through the workshop, as well as individual research articles in this special issue.

## Sufficiency and efficiency of phosphorus utilization

A sufficient (adequate for the purpose) and efficient (performing with the least waste of effort) utilization of P may offer a great reduction potential in animal husbandry and crop production (Withers et al. 2014). The management of animals, being an integral part of sustainable farming systems, plays a key role in reducing P inputs to soils and, consequently P losses from arable lands and grasslands. Because of the regional concentration of animal husbandry, improved diets with less P may be most urgently required and effective in regions with high stocking density. One area that attracted more research since the 7th IPW is related to animal performance and genetics. Studies with different poultry species found a heritability of between 0.10 and 0.22 for different criteria of P utilization (de Verdal et al. 2011; Beck et al. 2016), which points to the possibility of breeding for improved P utilization. Oster et al. (2016) found that, for pigs, genes involved in pathways relevant for P utilization were differentially expressed due to variable P supply and thus are potential candidate genes for improved P efficiency. In a forthcoming study, the impact of dietary P changes on growth performance (live weight, feed intake, feed conversion ratio), serum hormones (calcitriol, parathyroid hormone, triiodothyronine), bone characteristics (dry matter, crude ash), and transcript abundances of key players in re-absorbing and re-excreting tissues are reported. Transcripts associated with vitamin D hydroxylation (Cyp24A1, Cyp27A1, Cyp27B1) were regulated by diet at local tissue sites. Animals fed with low-P diets showed attempts to maintain mineral homoeostasis via intrinsic mechanisms, whereas the animals fed with high-P diets adapted at the expense of growth and development (Oster et al. 2018, this issue). A keynote lecture by Rodehutscord reported that feeding systems have been modified in ways that reduce P excretion without compromising animal health and performance. For dairy cows, mineral P supplementation of the feed is generally not necessary and might be needed only when fed with high amounts of corn products. Since IPW7, research in this area has been intensified with the aim of further reducing P excretion by livestock. This includes more precise prediction of the dietary P requirement and a better characterization of the availability of different P sources used in animal feed. With regard to nonruminants, much attention has recently been given to the variation in plant P sources, in particular phytate-P (Rodehutscord and Rosenfelder 2016). It has been known for about two decades that the use of the enzyme phytase as a feed additive can effectively increase phytate-P availability in pigs and poultry. New enzyme products and modifications of their level of inclusion in the diet have achieved phytate-P digestibility of up to 90% (e.g., Zeller et al. 2015). Consequently, the use of mineral P supplements and livestock P excretion can be reduced substantially.

Sophisticated analytical techniques like stable isotope-techniques (33-P, 18-O), NMR- and synchrotron-based spectroscopies are becoming more popular for quantifying P cycles, fluxes, and dynamics in the soil and other environmental systems. For instance, Tamburini et al. (2014) reviewed the usefulness, limits, and challenges of measuring the isotopic composition of oxygen within the phosphate ion, to improve our understanding of P cycling in soil and plant systems. From a process viewpoint, von Sperber et al. (2015) quantified the isotopic fractionation of O due to acid phosphomonoesterase and phytase on phosphate released by enzymatic activity. Furthermore, von Sperber et al. (2016) used raman spectroscopy to show how fast pyrophosphatase, another important enzyme, could completely exchange the oxygen atoms within the phosphate ion with oxygen atoms originally within water molecules. At the organism level, Pfahler et al. (2013) demonstrated that the isotopic composition of oxygen within phosphate ions in plant leaves was different from that observed in the solution delivering P to the plant. The authors explained this by the pyrophosphatase-mediated oxygen exchange between water and phosphate and by the activity of acid phosphatase releasing P from organic compounds in the leaf. Oxygen isotopes offer useful insights into the processes controlling P dynamics in terrestrial systems, especially the importance of enzymatic processes.

Analyzing the isotopic composition of oxygen bound to P (δ^18^O-P) is, however, constrained by a number of analytical difficulties (Tamburini et al. 2010, 2014; Frossard et al. 2011). The first one is to purify extracted PO_4_ from any possible contamination with other oxygen-containing compounds. At the end of this process, PO_4_ is precipitated as Ag_3_PO_4_ that is analyzed by temperature conversion, element analyzer, isotope ratio mass spectroscopy (TC-EA-IRMS). Another challenge is to quantify the possible exchange of O that could take place between phosphate and the solvent, e.g., during extraction with acids or the release of phosphate from organic P digestion under ultraviolet irradiation. Finally, the measure of δ^18^O-P can also be challenging as there are no internationally accepted standards for Ag_3_PO_4_. Answers on how to address these pitfalls to obtain meaningful results have been described in the above-mentioned publications.

The scientific discussion on the identification of soil organic P forms—whether soils contain simple well-identifiable organic P forms or organic P in complex macromolecular, nonidentified structures—is continuing. Using liquid state ^31^P NMR, McLaren et al. (2015) showed that high-molecular weight organic P fractions had much less distinct peaks than low-molecular weight fractions. Furthermore, Jarosch et al. (2015) showed that the proportion of high-molecular weight P was almost identical to the proportion of organic P that could not be hydrolyzed in the presence of different phosphatases. These results suggest that the high-molecular weight P fraction (i) can account for a large proportion of total organic P, (ii) presents structure that remains to be discovered, and (iii) is “resistant” to enzymatic hydrolysis.

Recent research supports the idea that the cycles of the biogeochemically important nutrient elements C, N, and P are closely interlinked across environmental systems. For instance, three long-term experiments, under differing conditions in Australia, Burkina Faso, and Switzerland, and subject to different types of organic (including manure and plant residues) or mineral fertilizer inputs, showed that the C-, N-, P-stoichiometry of soil organic matter was primarily controlled by soil properties rather than by the elemental stoichiometry of manure or fertilizer inputs (Frossard et al. 2016). In this context, a long-term field experiment running since 1998 in Northern Germany shows that organic P forms in soil did not correspond with the P forms in the organic fertilizers applied to the soil (Requejo and Eichler-Löbermann 2014). Although the study of C-, N-, and P ratios is needed to understand the long-term functioning of cropped soils, it must always be coupled with assessment of elemental inputs and budgets, and the ability of soils to stabilize C-, N-, and P-containing compounds.

A number of manuscripts were recently published on soil organic P mineralization and on factors controlling the plant–soil–microbes interactions. The methods used to assess soil organic P mineralization were reviewed by Bünemann (2015). The relative importance of P fluxes arising from soil organic matter (SOM) mineralization compared to fluxes from P desorption appears to be much larger in forest and grassland than in arable soils. Factors such as wetting and drying cycles, green manure inputs, seasonal fluctuations, and soil parent material also clearly affect organic P mineralization (Liebisch et al. 2014; Randriamanantsoa et al. 2015; Bünemann et al. 2016). Besides these quantitative approaches, functional genes coding for microbial alkaline phosphatases were analyzed. The genetic diversity of the phosphatases called PhoD (phosphodiesterase/alkaline phosphatase) and PhoX (alkaline phosphatase) was studied as well as environmental factors controlling this diversity (Ragot et al. 2015, 2017).

The application of microbial inoculants as so-called biofertilizers has often been described as a component of sustainable nutrient management. The main efforts in this field have focused on fungi (Vassilev et al. 2016; Ceci et al. 2018, this issue). However, the application of plant-growth promoting rhizobacteria (PGPR) has also been shown to increase nutrient availability in soil and to enhance plant growth by release of growth stimulating hormones or protection against soil-borne pathogens (Berger et al. 2015). The effects of biofertilizers can vary and failures of microorganisms to promote plant growth and increase soil nutrient availability have also been reported. Using a ^33^P-labeling approach Meyer et al. (2017) could not detect any significant effect of the strain *Pseudomonas protegens* CHA0 on soil P solubilization. Furthermore, soil inoculation with *P. protegens* CHAO slowed down soil respiration, suggesting that this strain, known for its antifungal activity, slowed down soil microbial activity and had little net effect on soil P availability. Considering the uncertainty and the costs of microbial inoculants in practical agriculture, the activation of native soil microorganisms by agronomic measures like organic matter management and crop rotation could be a better approach to utilize benefits of microbes (Tiemann et al. 2015; Hupfauf et al. 2016).

New secondary fertilizer products as well as improved P-fertilizer placement technologies have gained much attention in recent times (Brod et al. 2016). These new fertilizer products include ash, which is produced by the incineration of sewage sludge in the presence of chloride compounds to remove heavy metal contaminants. Results showed that sewage sludge ash treated with MgCl_2_ are very effective as P source and more suitable for plant P nutrition than CaCl_2_-treated ash (Nanzer et al. 2014a; Vogel et al. 2015, 2017). This was explained by the synthesis of Cl-apatite at high temperature during the treatment with CaCl_2_ (Nanzer et al. 2014b). Another secondary fertilizer product is struvite (magnesium ammonium phosphate), precipitated from liquid waste streams. Recently, several studies confirmed that recovered struvite is an effective P fertilizer under a wide range of crops and growing conditions (Vogel et al. 2015; Kataki et al. 2016; Talboys et al. 2016).

P-fertilizer recommendations are based primarily on an assessment of plant-available P concentrations in the soil (Jordan-Meille et al. 2012). Over the last few years, the limitations of many traditional methods to assess plant-available P in the soil have become increasingly apparent (e.g., Christel et al. 2016). These methods, based on batch extractions with various chemical extractants, represent the total static amount of P in the soil which is supposed to be plant available during the growing season. Comparative studies of the performance of various extraction procedures reveal that the range of extracted P (as a proportion of total P) varies widely depending on the extraction procedure. Moreover, inter-comparison of different extraction methods often showed only weak correlations between different methods. Consequently, results of different extraction methods can often not be directly compared (e.g., Shwiekh et al. 2015).

According to physicochemical considerations, the P dissolved in the soil solution should be immediately available for plant uptake. This amount, however, is exceedingly small and is constantly replenished by desorption from soil minerals. A relatively new method for predicting soil P availability which mimics this physicochemical process is the diffusive gradients in thin films (DGT) technique (Kruse et al. 2015; Christel et al. 2016). This technique is based on phosphate accumulation on a ferrihydrite-binding layer after passage through a hydrogel, which acts as a defined diffusive layer. It has been shown that P availability assessed with the DGT can be a better predictor for plant yield, compared with traditional soil tests (e.g., Six et al. 2013). However, the relatively low binding capacity of the DGT gel can be a problem for recently fertilized soils and high amounts of readily available P, and in this situation, DGT-binding gels with a greater P-sorption capacity are needed (Christel et al. 2016).

P-fertilization recommendations in most European countries are currently centered on the plant-available P content in the soil and the expected nutrient uptake by the crops (expected yield × P concentration of crop). P-fertilizer recommendations entail three steps (Jordan-Meille et al. 2012): (i) extraction of plant-available soil P, (ii) calibration of those soil test results, (iii) deducing recommended P-fertilizer amounts. Further, many additional factors besides plant-available P have an influence on the effectiveness of P fertilization, including soil properties, weather and climate, morphological and physiological strategies of crops, fertilizer type, and fertilizer placement (Jordan-Meille et al. 2012; Recena et al. 2016). However, these factors are not considered in traditional P-fertilizer recommendation schemes, and their assessment is, in most cases, time consuming and tedious.

The development and improvement of P-fertilizer recommendation schemes can profit from meta-analyses of long-term fertilization experiments (Kuchenbuch and Buczko 2011; Buczko et al. 2018, this issue). Such analyses have shown that optimum yields are attainable even for soil P contents which are deemed suboptimal according to P-content classes based on traditional extraction procedures, and that several soil and environmental parameters have an influence on the yield response of P fertilizers. Results of the more recent meta-analyses have not yet been considered in traditional P recommendation schemes and, too much P fertilizer has been and, in some countries in Europe, is still being recommended and applied, leading to P-balance surpluses and a build-up of legacy P in the soil (van Dijk et al. 2016; von Tucher et al. 2018, this issue).

In response to these undesired soil P enrichments, there are two key developments that need to be mentioned: (1) the now global acceptance of the “4R principles” of nutrient management (right place, right time, right form, and right rate) (Bruulsema et al. 2009) which have been updated to a strategic framework of “5R stewardship”(Re-align P inputs, Reduce P losses, Recycle P in bioresources, Recover P in wastes, and Redefine P in food systems) (Withers et al. 2015); and (2) nutrient-management-efficiency gains encompassed by the term “feed the crop not the soil,” which is being now taken up widely by the fertilizer industry as a driver for the development of new fertilizer products (Withers et al. 2015). In parts, these principles have been applied in certain countries like Switzerland (e.g., Flisch et al. 2009; Frossard et al. 2009); however, a wider acceptance and implementation is needed.

The further development of site-adapted cropping systems was mentioned as one of the challenges for the future agriculture during the 7th IPW in Uppsala (Sharpley et al. 2015). There is still the need to increase the overall P efficiency in cropping systems and to reduce P losses from agricultural fields. A new research focus on developing P-efficient cultivars that require less P (lower grain total P and lower phytate P) is described by Yamaji et al. (2017). Other measures for a better P efficiency of cropping systems should also include the application of diverse crop rotations, the extension of cover crop cultivation, and mixed cropping (Latati et al. 2014; Rose et al. 2016; Bakhshandeh et al. 2017). Mixed cropping and multispecies agroecosystems can result in an enhanced productivity compared to that of monoculture. The main advantage of mixed cropping is the efficient utilization of resources, such as light, water, and nutrients. This can be explained by complementarity and facilitation processes (Hinsinger et al. 2011). Combined cultivation of cereals and legumes is often practiced because of their complementarity in the use of N resources which may positively affect the yield and protein content of cereals (Mikic et al. 2015). Positive examples were also given for monocot fodder crops like maize and sorghum combined with legumes under suboptimal P supply (Eichler-Löbermann et al. 2016).

## Phosphorus recycling: Technologies and product applications

For the first time at an IPW meeting, there was a separate session dealing with current efforts and challenges for developing a circular P economy, with a focus on P-recycling technologies and product applications. Each year millions of tons of fossil P are mined and processed (USGS 2015), while the potential to recover and recycle waste P (in sewage sludge, manure, and food waste) remains untapped or inefficient. In recent years, various technical solutions have been developed as alternatives to traditional nutrient recycling routes and to allow more flexibility and more precise applications. These allow P recovery and provide renewable mineral compounds suitable as raw material for fertilizer production, or directly ready-to-use “renewable” fertilizer (e.g., Vogel et al. 2018, this issue), and even high-quality P products applicable in chemical and food industry, like phosphoric acid or the highly reactive allotrope white P (tetraphosphorous, P_4_).

**Fig. 2:**
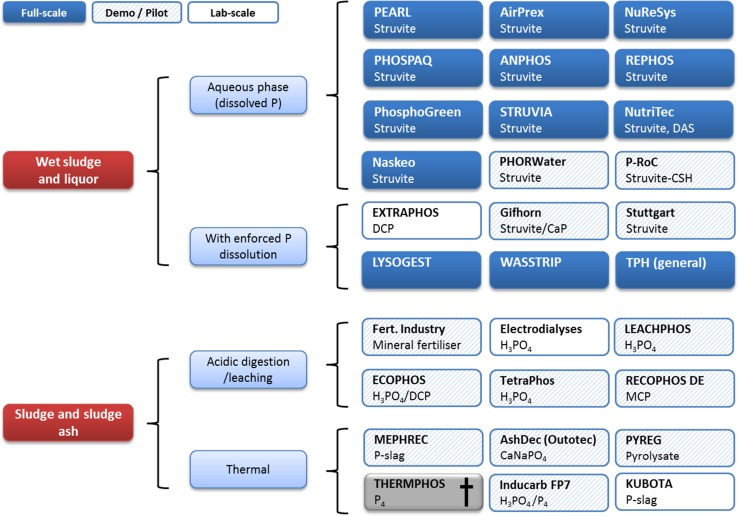
Overview on P-recovery technologies in Europe (Kabbe et al. 2015; Ohtake and Okano 2015; Kabbe and Kraus 2017)

However, in agricultural areas with a misbalance of P output and P demand, there is some need for P-extracting and -recycling technologies, e.g., for concentrated animal production on industrial scale and biogas plants “importing” their substrates from a larger agricultural area. Beside the agricultural byproducts, the wastewater treated in domestic wastewater treatment plants (WWTPs) is one of the most attractive, renewable P sources in the industrial world. On average, each person excretes daily about 1.5 g P worthy of being recovered. Consequently, in the recent years, a wide variety of technologies aiming at recycling the P in WWTP have been developed (Fig. 2). Technologically, they can be categorized into two principle routes: the recovery from solid and that from liquid phases of waste. Combinations including phase transfers between the both phases are considered as well.

Technologies which recover P from the liquid phase (e.g., struvite recovery or adsorption of dissolved phosphate by silica hydrates) provide operational benefits for wastewater treatment plants, such as prevention of unintended struvite precipitation in pipes and aggregates of the sludge treatment stream, improved sludge dewaterability for reduced sludge volume to be disposed of and reduced flocculation aid consumption. However, struvite recovery and similar technologies are quite limited in application range and recovery rate (5–25% without forced re-dissolution technologies). Since the prerequisites for their implementation are orthophosphate concentrations above 100 mg/L in the sludge water, only WWTP which removes P by enhanced biological P removal (EBPR) and subsequent anaerobic digestion (AD) can be seen as having market potential. On European or even global scales, struvite recovery is most often applied and represents the lowest hanging fruit in terms of alternative P-recovery and subsequent P-recycling options.

Due to the limited number of WWTPs operating EBPR + AD, and the limited recovery rates, technical solutions to remove the P from the sludge solids in WWTP by applying chemical P precipitation with iron (Fe) or aluminum (Al) salts are also needed. Most technologies operate by manipulating pH to acidic (P dissolution) and alkaline (P precipitation) conditions. A great challenge here is to minimize consumption of acids, bases, and further additives. One of these attempts is the EXTRAPHOS^®^ process recently developed by the Chemische Fabrik Budenheim KG, 55257 Budenheim, Germany. Here, the P is recovered on-site at the WWTP as Dicalciumphosphate (DCP) after extraction from digested sludge with CO_2_ under pressure (Schnee and Stössel 2014). At current price levels for phosphate rock and other raw materials, none of these technologies could be implemented profitably, unless the technologies provide other benefits to operators than just nutrient recovery.

Since undiluted incinerated sludge provides the highest mineral concentrate within the sewage sludge stream, P recovered from mono-incineration ash represents a promising P concentrate after the wastewater treatment. There is an obvious trend that most technologies at the brink to the market nowadays intend to yield phosphoric acid as commercially viable product, as represented by ECOPHOS (de Ruiter 2014) or TetraPhos (Hanßen et al. 2016). Another ambitious technology is the production of white P (P_4_) out of ash by thermochemical conversion (RecoPhos FP7, ICL; Langeveld 2016).

Also the recovery and recycling of agricultural waste have been widely discussed during IPW8, for example, digestate, process animal manure, compost, or biochar. The advantages of pyrolysis include the significant reduction of the raw material, the destruction of pathogens, and the formation of a carbon-rich substrate (Bünemann et al. 2016). Research has mainly focused on optimization of the pyrolysis process, understanding the binding mechanisms of P and other constituents and on the P availability of the product for plant growth (Robinson et al. 2018, this issue).

The steel industry represents an important P consumer and P-waste producer. In Japan, the P flow in waste steel slag is 104 kt/a, which is nearly the double the P imported for fertilizer (53.3 kt/a) or human P excretion (55.9 kt/a) (Matsubae et al. 2011). Basic slag from the Thomas process has been used as fertilizer for about 70 years, because it contained a high P_2_O_5_ content of 10–15% w/w. However, the Thomas process had been replaced by other smelting processes using iron ores low in P. A large research and development project is currently been conducted by two leading steel companies in Japan to recover P from P-rich steelmaking slag, called dephosphorization slag (Ohtake and Okano 2015; Ohtake et al. 2018, this issue). Importantly, the rest after recovering P still contains valuable resources such as iron, calcium, and silicates. The P recovery from steelmaking slag aims to recycle the rest as raw materials to the blast furnace, thereby improving the resource efficiency of the steelmaking process. Meanwhile, steelmaking slag can also be used as recyclable calcium silicates to prepare a bifunctional adsorption–aggregation agent for a simple phosphate recovery technology (Ohtake et al. 2018, this issue).

**Fig. 3:**
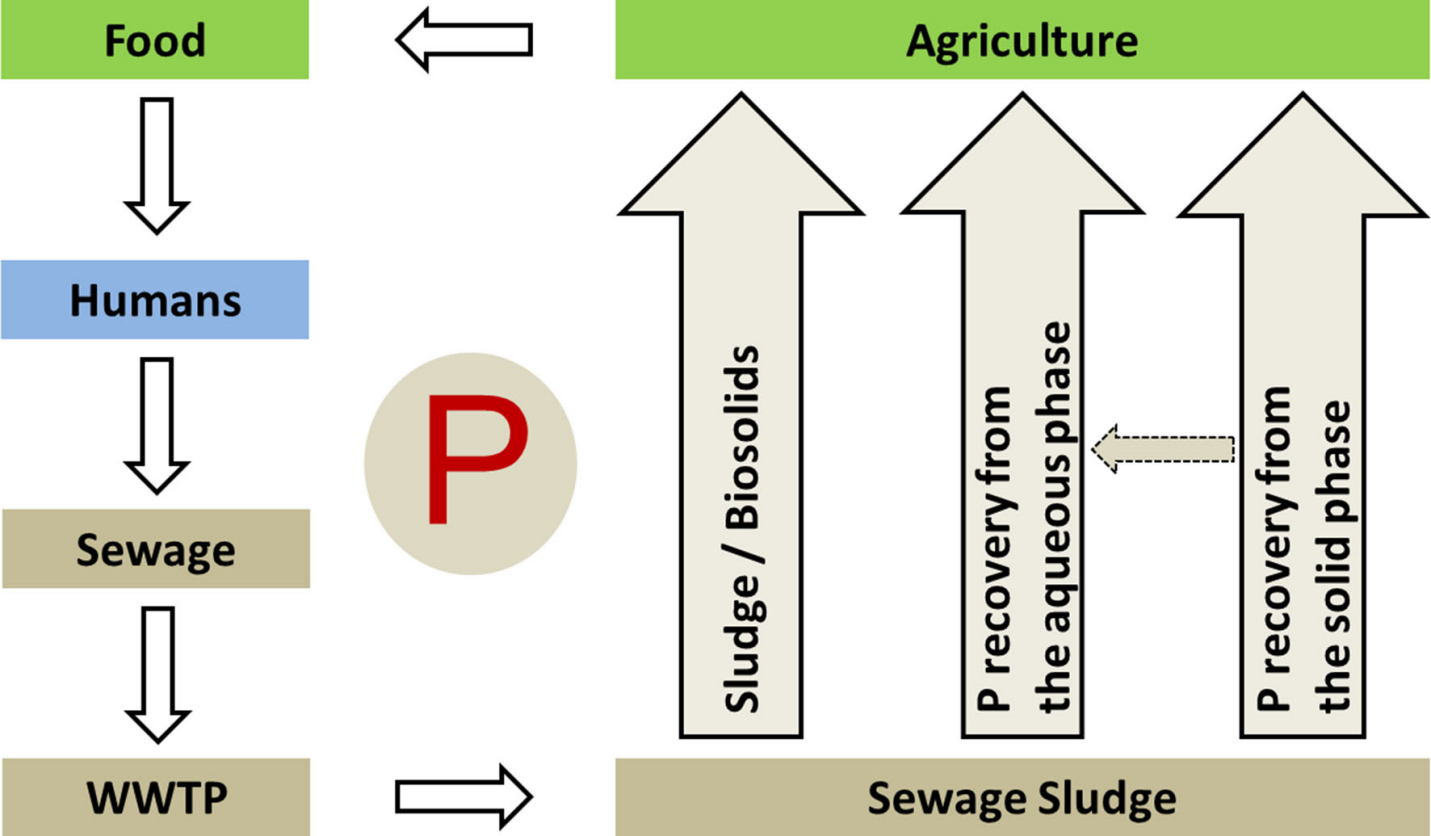
Three principal routes for nutrient recycling from sewage to agriculture (Kabbe 2013)

Nutrient recycling only happens if the recovered nutrients are returned into the nutrient or production cycle, as fertilizer or feed, as food or fodder additive, or as products of the chemical industry. Since industrial demand for P is small compared with agricultural use, and industrial applications demand higher-quality P products, it is likely that the majority of recovered P will and should be recycled as fertilizer or in animal husbandry, thus directly closing P cycle for food production (Fig. 3).

Although several countries (e.g., Germany, Switzerland) have opted to strengthen P recovery by legislation, the recycling question still remains unsolved. Various regulations interfere with the nutrient recycling and somehow discriminate or even exclude nutrients recovered from renewable resource from recycling. Fertilizer may serve as an example: During IPW8, it was stated that recovered P products still cannot compete economically with P from fossil sources. A first step toward a real recycling should therefore be to provide a level playing field for all fertilizers, irrespective if they are made of fossil or of secondary sources. Otherwise, legal obligations to recover would fail because of missing options for commercialization. This has to be flanked by the definition of End-of-Waste criteria, i.e., quality criteria for waste-based materials to be destined for recycling as secondary raw materials or even products, for recovered nutrients and by binding recycling targets comparable to the CO_2_ emission reduction goals. Otherwise, neither recovery nor recycling technologies will be widely implemented except under those conditions where they provide operational or monetary benefits to its operators by operational side effects.

Nutrient recovery is still lacking a demand-side market pull for recovered (secondary) nutrients, and the biggest challenge will be bridging the gap between supply (recovery) and demand (recycling). Whereas in the past, the focus of nutrient recovery technologies was merely on high recovery rates for single nutrients, now energy efficiency, synergies, and cost have become more and more important. Resource efficiency can never be tackled for just one nutrient alone; thus, the link to the other macronutrient element nitrogen (N) will be an important goal.

The value chain up- and downstreams of the P recovery is poorly addressed so far (Schoumans et al. 2015). Upstream, prevention of P-containing waste is a societal challenge. At least, waste should be generated in forms that support downstream recovery. Recovery neglecting the settings and requirements of downstream recycling will remain only a theoretic option. Only integrative solutions will provide the chance for sustainable implementation. In this context, future research should strive even stronger while seeking for holistic solutions.

## Phosphorus fluxes and cycling in the environment

Elevated P inputs can have severe long-term effects on freshwater and marine ecosystems, and large-scale efforts are needed to reduce P inputs from land. In the case of the Baltic Sea, P inputs from the drainage basin have been reduced since the early 1990s, i.e., by 20% between 1994 and 2010 (HELCOM 2015), and progress toward improved environmental status in some parts of the Baltic Sea has been observed (Nausch et al. 2016; Andersen et al. 2017). However, eutrophication is still considered to be the most serious anthropogenic threat in the Baltic Sea, and the P target values of the Baltic Sea Action Plan (BSAP) have not yet been reached: max. allowable input is 21 716 t by 2021. P input from the drainage basin was 36 200 t in 2010 (HELCOM 2014, 2015). The slow recovery of the environmental status of the Baltic Sea is also a consequence of the long residence times and the large quantities of P stored and recycled in sediments (e.g., Leipe et al. 2017), although mobilization from marine sediments in a shallow oxic lagoon was found to be relatively small (Berthold et al. 2018, this issue).

The mitigation of eutrophication in freshwater, coastal, and marine systems requires a better understanding of mobilization and release of P from soil and catchments (soil-to-water transfers), P composition and cycling in water bodies, and measures to decrease P loss. New findings reveal that, in some river basins, P export now exceeds P inputs, which may result from the net mobilization of P pools accumulated during earlier decades (Powers et al. 2016). This appears to occur mainly in agricultural and mixed-used catchments (e.g., in UK and USA), which have been under fertilizer treatment for several decades, and which may have already reached a finite P-accumulation stage. In other areas (e.g., China), where P fertilization has been introduced only one or two decades ago, P is still accumulating in soils (Powers et al. 2016). Accounting for, and predicting, the long-term impacts of P cycling from legacy stores were highlighted as a key future challenge in the IPW7 Synthesis paper (Sharpley et al. 2015). Over the last 2 years, there has been growing interest in utilizing long-term historical datasets (e.g., Rupp et al. 2018, this issue) and reconstructing historical watershed P balances to evaluate trajectories of legacy P accumulation and drawdown in catchments (e.g., Liu et al. 2016; Powers et al. 2016). Moreover, there have been developments in monitoring, measurement, and process studies to estimate legacy P contributions to contemporary catchment P budgets (e.g., Liu et al. 2015; Waldrip et al. 2015), and also in identifying opportunities to incorporate the utilization of legacy P within sustainable P-management strategies (Rowe et al. 2016).

The mobilization and transport of P at the catchment scale are strongly influenced by weather/climate changes causing temporal and spatial differences in P losses (Mellander et al. 2015). Single rainfall events are of high importance for the annual P load and thereby revealing the threshold character of P mobilization as well as sediment-transfer processes (Ockenden et al. 2016, River and Richardson 2018, this issue). High rainfall and overland flow intensities provide the kinetic energy necessary to generate the critical shear stress for mobilizing soil particles and inducing sediment and sediment-bound P fluxes. There is increasing evidence that particle-bound P transport is not only limited to the soil surface, and that colloid-related transport through the soil profile can drive P losses (Sharma et al. 2017). Especially when dry periods precede high intensity rainfall events, (colloidal) P may be transferred over long distances via subsurface flow (Zhang et al. 2016). The travel distances of P-laden microparticles within a soil profile can be equivalent to those of dissolved compounds (Koch et al. 2016). Although agricultural P loading had been considered a surface runoff-dominated process, over the last 2 years, there have been new insights into the role of tile drainage in transport of both particulate and soluble P fractions (e.g., Smith et al. 2015a; King et al. 2016; Ulén et al. 2016). Tile drainage increases critical source areas as well as providing conduits for P loss that by-pass a large proportion of the P-sorbing soil matrix, and the contribution of subsurface drainage to river P fluxes in under-drained catchments may have been underestimated (Smith et al. 2015a). It is also expected that, in lowland catchments, tile-drainage provides a preferential pathway for P carriers, for instance, as ochre flocs consisting of Al, Fe, and Mn components with attached microorganisms (Zimmer et al. 2016).

P concentrations in river sediments are often high in comparison to the soils in the catchment (Pulley et al. 2016). A variable water flux with temporary high intensities may induce re-suspension of particulate P from the channel bed increasing total P (TP) loading of streams (van der Grift et al. 2016). As a consequence, P concentrations may increase along the flow path and spatial scale, from small ditches to the river basin outlet even without additional P sources such as effluent from sewage plants.

The effect of catchment hydrology on P export is P-component specific. It has been observed at mid-size river basin scale (3300 km^2^) that the dissolved reactive P concentrations decrease with the increasing discharge (daily resolution), whereas the TP concentrations tend to increase at higher discharge rates (Nausch et al. 2017). The soluble P fraction in surface waters is probably diluted by less P-laden groundwater, whereas TP dynamics, which include the sediment-bound P phase, reflect erosion processes (Nausch et al. 2017).

The expansion in the use and application of in situ high-resolution sensors over the last two years have resulted in key advances in P monitoring in freshwaters (Blaen et al. 2016; Rode et al. 2016). These technologies are providing new opportunities to evaluate the dynamics in P sources and pathways (e.g., Bowes et al. 2015; Lloyd et al. 2016; Mellander et al. 2016; Ockenden et al. 2016), the processing and cycling of P, linked to biological dynamics (Halliday et al. 2015; Cooper et al. 2016) and for identifying the effects of P relative to other multiple pressures on algal blooms and crashes (Bowes et al. 2016). Our understanding of spatial scale dependent P transport is limited; for example, the spatial variation in P leaching via subsurface drains can be higher than the temporal variation (Ulén et al. 2018, this issue). This requires deployment of spatially distributed and nested intelligent sensor networks which directly measure the dynamics in water and nutrient fluxes at time scales aligned with the variation of the physical drivers (Rode et al. 2016).

New efforts are also underway to investigate innovative P-mitigation and -removal measures and to improve existing measures such as constructed wetlands (e.g., Geranmayeh et al. 2018, this issue). For example, P removal in large wastewater treatment plants has been improved, but in some countries such as Germany, small rural wastewater treatment plants were neglected; they do not even have legally binding P emission limits. Most recently, their emissions have been investigated, and new solutions of efficient P removal are being developed (Cramer et al. 2018). Mitigation strategies for controlling P export to rivers and coastal systems should include the regulation of water flows such as controlled drainage (Young et al. 2017). Controlled drainage, a frequently discussed P-attenuation approach in lowland catchments, can lower the P load released to surface water bodies because of a reduction in water flux (Zhang et al. 2017). However, elevated P concentrations in groundwater may counteract the positive effect of controlled drainage (Rozemeijer et al. 2016). In catchments where P concentrations increase along the flow path, soluble P attenuation and particulate P mobilization should be maximized and minimized, respectively, for example, by cleaning out the sediments before they become saturated with P and encouraging vegetation growth on ditch beds (Shore et al. 2016).

There is also growing interest in the long-term water-quality impacts of agricultural management and conservation practices, and indications that, over time, some conservation practices aimed at reducing particulate P losses, may have unintended tradeoffs for soluble P losses (Jarvie et al. 2015, 2017; Dodd and Sharpley 2016). This is of particular current concern in the western Lake Erie basin (WLEB), U.S., which has undergone a marked phase of re-eutrophication, linked to increased soluble P fluxes (Smith et al. 2015b; Jarvie et al. 2017). Within the WLEB, there have been long-term, large-scale changes in land management: conservation tillage to minimize erosion and particulate P loss, and increased tile drainage to improve field operations and profitability. The increased soluble P fluxes, water-quality impairment, and harmful algal blooms within the WLEB exemplify much wider challenges for P and water-quality management, as a result of the convergence of multiple pressures and potential tradeoffs in agricultural management practices, compounded by climate change (Smith et al. 2015b; Williams et al. 2016; Jarvie et al. 2017) and highlight the need for adaptive management and optimizing land use and management to address tradeoffs and reduce water-quality impairment (Doody et al. 2016; McDowell et al. 2016).

Questions remain especially regarding the consequences of these new findings for the design of P-retention measures: What is the impact of individual events on P leaching and what is the implication for mitigation measures? What measures can be undertaken to reduce the P-loss risk from soil, considering that strategies, such as buffer strips and modified tillage practices which yield great benefits in some settings, can lead to tradeoffs and adverse effects in other situations? Here, there is growing recognition that beneficial management practices need to be spatially and temporally precise and tailored to address the site-specific characteristics of the land, climate, and farming system. How can the P fluxes through drainage tiles be addressed? What measures can remediate high P emissions from groundwater? How can new emission sources be detected and defined? How to deal with the difficulty of many small P-emission sources within a catchment, hence many different measures, resulting in numerous conflicts with landowners and high costs? How are measures working in the long run? Mitigation measures have long time lags until improvements become apparent in lakes and coastal waters.

## Phosphorus governance

For the first time in the history of the IPW series, a whole session was dedicated to the topic of phosphorus governance from the perspective of social sciences. In this session, there were contributions from diverse backgrounds, including economics, political and social sciences, and law, to explore the political and legal instruments needed to close nutrient cycles and to sustain natural resources. Special concern was given to measures aiming at reducing consumption of animal products.

Closing nutrient cycles in agriculture at certain spatial scales and environmentally sound levels, increasing nutrient recovery and fertilizer efficiency clearly belong to the political agenda. Indeed, strategies are needed to reduce the heavy environmental and resource impacts associated with the current industrialized agricultural systems. However, it is doubtful, whether these consistency and efficiency strategies alone will be sufficient in order to achieve certain environmental goals set at the global level: Art. 2 (1) of Paris Agreement sets out the objective to keep the increase in global average temperature to well below 2 °C or even 1.5 °C above pre-industrial levels. Furthermore, Art. 1 of the Convention on Biodiversity (CBD) requires the ‘conservation’ of biodiversity. If these ambitious objectives are taken seriously, it rather seems to be necessary to complement technological measures with supplementary (voluntary or mandatory) sufficiency measures. This would particularly address the food sector and more specifically the production and consumption of animal products.

When considering the implementation of efficiency, consistency, and sufficiency strategies, experiences made with the effectiveness of certain groups of instruments need to be taken into account. Especially regulatory command and control measures are subject to considerable doubts. The potential weaknesses of regulatory command and control approaches become particularly apparent in the area of agriculture:

- *Enforcement* Regulations in the area of agriculture often suffer from weak enforceability. Despite the unused potential to improve the efficiency of enforcement, there is no fully satisfactory solution to the problem, that a strong enforcement would require the monitoring of a nearly uncountable number of agricultural activities.
- *Shifting effects* Reducing fertilizer use locally within the EU or one EU member state might lead to a stronger transfer of agricultural production abroad. A merely regional or local reduction of fertilizer use is thus prone to shifting effects, without guaranteeing an absolute reduction of emissions or resources used at the global scale. While not excluding the use of regulatory instruments in principle, the risk of shifting effects demonstrates the importance of at least regionally and ideally globally concerted actions.
- *Rebound effects* Reducing the average nutrient input per plant does not prevent overall land-use increases due to the production of fodder or energy crops. Therefore, the reduction of emissions and resource-overuse from one sector might easily be (over)compensated by undesired developments in other sectors. Rebound effects prevent the achievement of absolute reductions.

In fact, behavioral sciences (Scholz 2011; Stoll-Kleemann 2014; Stoll-Kleemann and O’Riordan 2015; Ekardt et al. 2015; Ekardt 2016) can explain, why not only politicians, farmers, and the fertilizer and food industry, but also consumers are indeed not sincerely interested in effectively addressing adverse ecological impacts of agriculture. This is not only due to the frequently mentioned lack of information or cost–benefit considerations of single actors (not discussed in the IPW7 summary by Sharpley et al. 2015). Instead, the causes lie much deeper: in particular regarding the consumption of animal products, a central role must also be assigned to concepts of normality, comfort, habits, repression, search for recognition or the tendency to forget about cognitive dissonances.

These insights make clear, that the environmental challenges ahead cannot be solved with simple adjustments of existing instruments. These motivational problems and systematic weaknesses of regulatory instruments rather call in favor of a policy approach that addresses absolute quantities of resources and pollutants. In the light of the level of ambition set by global environmental goals, such a quantity control would need to pursue efficiency and consistency strategies, but would also need to tap into the potential of sufficiency (not discussed in the IPW7 summary by Sharpley et al. 2015).

A first and rather easy step of quantity control would be the reduction of agricultural subsidies, as this would help to reduce the associated problems of overproduction. This alone might, however, not be ambitious enough. Instead, it would be central to reduce the quantity of fossil fuels used. Fossil fuels are the key factor for several, closely interconnected environmental problems. Data from the IPCC suggest, that, in order to stay well below the 2 °C or even 1.5 °C objective, fossil fuels would need to be retrieved from the market by 2027 (1.5 °C) or 2038 (< 2 °C). Such a radical reduction in the use of fossil fuels could be implemented by an EU emission-trading scheme that would cover all fossil fuels, include all emissions from livestock farming and would be combined with a border adjustment for imports and exports. A drastically reduced consumption of animal products, biofuels, and a reduction of food waste would be a likely consequence. By this means, a variety of adverse impacts could be addressed simultaneously: biodiversity loss, degradation of soils, nutrient water pollution, CO_2_ emissions from the transport of agricultural products as well as emissions of ammonia and nitrous oxide from manure management and fertilized soils. As mineral fertilization today is often based on the use of combined NPK fertilizers, a reduced mineral fertilization in general would also conserve scarce P resources. By the same token, a reduced consumption of livestock products could improve global food security, since it provides the potential to leave more calories to the poor (albeit undernutrition has several reasons; generally speaking, neither global food security nor geopolitical issues were the major topics of IPW8).

However, a quantity control of fossil fuels alone does not address issues due to all greenhouse gases (Ekardt et al. 2015; Ekardt 2016). It might also partly frustrate the important objective of land-use reduction, which is particularly important for the conservation of biodiversity. This tradeoff could be partly avoided by including livestock in an emission-trading scheme. However, supplementary measures could be necessary. It could be considered to either control the quantity of agricultural land or the P used. The pricing of agricultural land as such, via taxes or a trading of land certificates, would address the various agricultural (greenhouse gas and ammonia) emissions and would help to reduce the pressure on land. If a tax on agricultural land would be progressive, small-scale farming and organic farming, having sometimes environmental and resource benefits, would be promoted.

Pricing P from primary fossil resources could lead to a reduction of fertilization. A price (by means of charges etc.) on mineral P might also help to close disrupted nutrient cycles not only at the farm level, but also by fostering recycling from P from sewage and waste. Because of the potential contamination of secondary P resources, this also requires effective waste treatment technologies, as well as legal standards in order to prevent soil contamination. The problem of contamination illustrates that even a comprehensive approach of quantity control must be complemented by classical regulatory instruments, such as environmental and technical standards. In particular, hot-spot problems such as the local accumulation of contaminants, nutrient surpluses, or the need to protect particularly sensitive local ecosystems demonstrate that an approach, including both quantity control and regulatory standards, is necessary. These issues of instrument mix and interrelated environmental problems are also new compared to the IPW7 summary as well as the broader analysis of behavioral factors and governance deficits discussed above.

## Summary and conclusions

Managing the scarcity and necessity of P requires an adapted agriculture, i.e., adequate animal feeding and fertilization of crops. In animal husbandry, recent research has opened up new opportunities for reducing the P added in feed by exploring the genetic potential of farm animals, adapted feeding systems, and improved phytate-P digestibility, to reduce P excretion by animals. In soil-based cropping systems, sophisticated techniques of analysis, such as the ^18^O-exchange technique, nuclear magnetic resonance (NMR), and synchrotron-based P-speciation techniques, are providing new opportunities to quantify the behavior, fate, and fluxes of P derived from manure and fertilizer sources. The roles of microorganisms, organic forms of P, and interlinked C-, N- and P-transformations were found to be more important than previously assumed, and this knowledge enables efficient P uses in growing agricultural crops. Nevertheless, meta-analyses of fertilizer experiment data have indicated that P-application rates are still too high, e.g., in Germany where reductions may be possible without crop yield losses. There are opportunities to use new soil testing methods (e.g., diffusive gradients in thin films, DGT) to develop new P-fertilizer recommendations. Furthermore, examples were given recently for site-adapted cropping systems involving catch crops, combined cultivation, or mixed cropping that could result in a better utilization of soil and fertilizer resources. Many new technologies have been developed in recent years for recovering P from liquid and solid phases of waste. Some of these, like struvite recovery, have already been upscaled to practical use and the products successfully tested in fertilization or other experiments. It was emphasized, however, that prevention of P-containing waste and recycling needs to be combined in holistic solutions which are actually not yet developed or tested.

Such improvements in agriculture toward a more sufficient and efficient P use very likely reduce the burden of P in aquatic ecosystems, although short-term improvements may be rather unlikely. Advances in process-understanding of the P burden have been made in disclosing the effects of weather/climate and hydrology on the export of P components from catchments. New P-mitigation and -removal measures have been described, but there is also new indication that conservation practices designed to reduce particulate P losses, when combined with other land-management practices such as broadcast fertilizer applications, may have unintended tradeoffs for soluble P losses, especially in the long term. This calls for long-term monitoring schemes and adaptive management at the landscape level. Mitigation of eutrophication in freshwater, coastal, and marine systems requires a better understanding of factors controlling P delivery from terrestrial systems as well as the understanding of internal processes such as recycling of P from sediments. Discussions of governance options pointed out that the above “technical approaches” probably will not solve the P problem. This has been discussed in the broader frame of international agreements like the Paris Climate Agreement and the Convention on Biodiversity. If these international agreements shall be fulfilled, general reductions are required in the total use of fossil fuels and P fertilizers as well as in the consumption of animal products. Governance approaches to achieve these goals involve the reduction of agricultural subsidies, caps for fossil, and nonrenewable fuels and fertilizers or pricing of these inputs to agriculture.

In summary, this special issue of *Ambio* compiles a multitude of new findings in agricultural and environmental P research, which are translated into governance options for a more sustainable P use. Altogether, this publication provides detailed and recent approaches to solve the P and related environmental problems, demonstrating the research progress in the period after the previous International Phosphorus Workshop.
